# Innate Immunity in the Middle Ear Mucosa

**DOI:** 10.3389/fcimb.2021.764772

**Published:** 2021-10-29

**Authors:** Helen M. Massa, Kirsten M. Spann, Allan W. Cripps

**Affiliations:** ^1^ School of Pharmacy and Medical Science, Griffith University, Gold Coast, QLD, Australia; ^2^ Centre for Immunology and Infection Control, School of Biomedical Sciences, Queensland University of Technology, Brisbane, QLD, Australia; ^3^ Menzies Health Institute Queensland, School of Medicine, Griffith University, Gold Coast, QLD, Australia; ^4^ School of Medicine and Dentistry, Griffith University, Gold Coast, QLD, Australia

**Keywords:** innate immunity, otitis media, non-typeable *Haemophilus influenzae*, influenza A virus, otopathogens, bacteria, viruses, mucosa

## Abstract

Otitis media (OM) encompasses a spectrum of clinical presentations ranging from the readily identifiable Acute OM (AOM), which is characterised by otalgia and fever, to chronic otitis media with effusion (COME) where impaired hearing due to middle ear effusion may be the only clinical symptom. Chronic suppurative OM (CSOM) presents as a more severe form of OM, involving perforation of the tympanic membrane. The pathogenesis of OM in these varied clinical presentations is unclear but activation of the innate inflammatory responses to viral and/or bacterial infection of the upper respiratory tract performs an integral role. This localised inflammatory response can persist even after pathogens are cleared from the middle ear, eustachian tubes and, in the case of respiratory viruses, even the nasal compartment. Children prone to OM may experience an over exuberant inflammatory response that underlies the development of chronic forms of OM and their sequelae, including hearing impairment. Treatments for chronic effusive forms of OM are limited, with current therapeutic guidelines recommending a “watch and wait” strategy rather than active treatment with antibiotics, corticosteroids or other anti-inflammatory drugs. Overall, there is a clear need for more targeted and effective treatments that either prevent or reduce the hyper-inflammatory response associated with chronic forms of OM. Improved treatment options rely upon an in-depth understanding of OM pathogenesis, particularly the role of the host innate immune response during acute OM. In this paper, we review the current literature regarding the innate immune response within the middle ear to bacterial and viral otopathogens alone, and as co-infections. This is an important consideration, as the role of respiratory viruses as primary pathogens in OM is not yet fully understood. Furthermore, increased reporting from PCR-based diagnostics, indicates that viral/bacterial co-infections in the middle ear are more common than bacterial infections alone. Increasingly, the mechanisms by which viral/bacterial co-infections may drive or maintain complex innate immune responses and inflammation during OM as a chronic response require investigation. Improved understanding of the pathogenesis of chronic OM, including host innate immune response within the middle ear is vital for development of improved diagnostic and treatment options for our children.

## Introduction

Otitis media (OM) is defined as inflammation of the middle ear and encompasses a range of clinical presentations. Acute OM (AOM) is characterised by otalgia and fever and may occur occasionally, particularly during a child’s first 3 years of life. If acute episodes occur a minimum of 3 times in a 6 month period or more than 4 times within a 12 month period the condition is considered to be recurrent (RAOM) (Granath, 2017). Otitis media with effusion (OME) is defined by the presence of middle ear fluid, or effusion, without any symptoms except impaired hearing due to restricted mobility of the tympanic membrane. Chronic suppurative OM (CSOM) involves perforation of the tympanic membrane with persistent fluid discharge for more than 6 weeks (Kong and Coates, 2009; Lieberthal et al., 2013; Massa et al., 2015; Bhutta et al., 2017).

The pathogenesis of OM is largely due to activation of the innate inflammatory responses to viral and/or bacterial infection of the upper respiratory tract (Mandel et al., 2008; Bakaletz, 2010; Mittal et al., 2014a; Parrish et al., 2019). Pathogen-induced inflammation in the nasopharynx (primarily due to viral infections) and eustachian tubes (both viral and bacterial infections) then leads to a range of responses including enhanced mucus secretion (Val et al., 2018), neutrophil extracellular traps (Thornton et al., 2013), damage to the epithelium and enhanced commensal bacterial colonisation (Nokso-Koivisto et al., 2015; Chonmaitree et al., 2016). Nasopharyngeal inflammation also causes a loss of pressure equilibrium with the middle ear (Martin et al., 2017), allowing fluid accumulation and invasion of viruses and commensal bacteria. Unresolved inflammation then leads to the reoccurring or chronic infections and fluid build-up in the middle ear that are the hallmarks of recurrent and chronic forms of OM (Bhutta et al., 2017).

In most children, AOM is resolved by the mucosal immune response of the middle ear and upper respiratory tract, that protects against repeated infections of the middle ear during the early years of development when children are at highest risk for ear disease. Progressively, the mean frequency of AOM episodes experienced by a child falls from 1.97 per year at six months of age to 1.07 per year by 36 months of age (Chonmaitree et al., 2008). Although overall, by three years of age, 60% of children have experienced one or more episodes of OM and 24% have experienced three or more episodes (Kaur et al., 2017). However, the global burden of disease caused by recurrent and chronic forms of OM, that are not as well controlled by the immune system, is considerable, particularly during the first 5 years of life. Globally, CSOM occurs in 4.76% of the population (22% in children under 5 years old) with hearing impairment present in 30 children per 10,000 (Monasta et al., 2012), a prevalence that may rise further in adults (Chung et al., 2016).

Our understanding of the role of the innate immune response in OM is improving as more evidence comes to light that frequent or prophylactic antibiotics are not always effective for chronic or recurrent forms of OM, or even for non-severe AOM, where a “watch and wait” approach to treatment is recommended (Lieberthal et al., 2013). In many clinical practice guidelines, antimicrobial decision-making is based on the clinical severity of AOM (Spoiala et al., 2021), with a previous randomised control trial in high-risk individuals reporting that long-term antibiotics did resolve OME and prevent AOM with perforation (Leach et al., 2008). However, for chronic forms of OM, such as CSOM, the benefits of either systemic or topical antibiotics for rapid resolution are not clear (Chong et al., 2021), and analgesics rather than antibiotics or anti-inflammatories are recommended as the front line treatment for OME (Rosenfeld et al., 2016; Chong et al., 2021).

The innate immune system is a critical first line of defence within the middle ear and is activated in response to pathogen associated molecular patterns (PAMPs) on invading pathogens (Li et al., 2013; Massa et al., 2015). Key characteristics of this defence mechanism include physical epithelial barriers, recognition of non-self-factors such as pathogens, up-regulation of the complement system, initiation of generalised inflammatory responses and activation of specific immune responses to pathogens through upregulation of the adaptive immune system (Massa et al., 2015; Preciado et al., 2017).

## Bacterial and Viral Otopathogens

Historically, the middle ear has been considered a sterile environment (Kurono et al., 1996; Lim et al., 2000). However, recent advances in ‘OMICS technology have revealed the potential of a middle ear microbiome (Marsh et al., 2020). The mechanisms by which the middle ear microbiome, in concert with that of the nasopharyngeal microbiome, may impact on the development and pathogenesis of OM remain largely unknown. This is a growing area of investigation (Schenck et al., 2016; Enoksson et al., 2020; Marsh et al., 2020), as the presence of a healthy middle ear microbiome is controversial (Johnston et al., 2019) with one key study in adults finding no evidence of bacterial colonisation of the middle ear using microscopy and culture techniques (Jervis-Bardy et al., 2019). The inflammation that defines OM, however, is induced by invading respiratory viral pathogens and dysregulated commensal bacterial populations within the nasopharynx, which then migrate through the upper respiratory tract *via* the eustachian tubes to infect the middle ear. How well these infections are then controlled by host mucosal immune responses, and the nature of each individual’s immune response, direct the course of OM disease.

Three bacterial species, *Streptococcus pneumoniae*, non-typeable *Haemophilus influenzae* (NTHi) and *Moraxella catarrhalis* are the dominant bacterial otopathogens globally (Schilder et al., 2016) although individual species and strain dominance may vary according to geographical location (Ngo et al., 2016). These bacteria are commensal in the nasopharynx and typically do not induce localised inflammation or activation of innate immune responses, however their presence within the middle ear stimulates both responses and may result in clinical presentation of AOM.

Functional, effective clearance of bacterial otopathogens from the middle ear, particularly for children experiencing RAOM and COME, may be disrupted by the presence of bacterial biofilm development on the epithelium of the middle ear (Hall-Stoodley et al., 2006; Thornton et al., 2011). Mucosal biofilms result from host and bacterial interactions, often multiple bacterial species (Thornton et al., 2011), incorporating host and bacterial DNA to develop and stabilise the biofilm. Furthermore, in addition to biofilms, the presence of intracellular bacteria, within the mucosal epithelial cells may also contribute to OM persistence and ineffective clearance of infection through antibiotic use (Bakaletz, 2012; Coates et al., 2008).

Viruses play an important role in the induction of AOM, which often occurs as a complication of upper respiratory infection (URTI) (Pettigrew et al., 2011). Some viruses can cause AOM in isolation, although most cases of AOM are the result of polymicrobial infections (Ruohola et al., 2013; Sawada et al., 2019). All respiratory viruses have been associated with AOM, in that they have been detected in middle ear fluid (MEF) or by nasopharyngeal swab during an episode of AOM, although some are more commonly detected than others (Pettigrew et al., 2011; Sawada et al., 2019). These include rhinovirus (RV), respiratory syncytial virus (RSV), adenovirus (AdV), human metapneumovirus (HMPV), influenza A virus (IAV) and seasonal human coronaviruses (Heikkinen et al., 1999; Chonmaitree and Heikkinen, 2000; Chonmaitree and Henrickson, 2000; Heikkinen and Chonmaitree, 2003; Kalu et al., 2011; Nokso-Koivisto et al., 2015; Chonmaitree et al., 2016; Schilder et al., 2016). In many studies, rhinovirus is the most common virus detected in both MEF and the nasopharynx of children with AOM (Moore et al., 2010; Yatsyshina et al., 2016), while in other studies RSV is most commonly detected (Sawada et al., 2019). Some viruses, however, are considered more “otopathic” than others, in that they are more likely to cause AOM without bacterial co-infection. A study that investigated the seasonality of AOM identified that peak AOM activity was significantly associated with detection of RSV, HMPV and IAV (Stockmann et al., 2013). RSV in particular is known to induce AOM in the absence of bacterial infection of the middle ear (Ruohola et al., 2013; Yatsyshina et al., 2016). There is substantive clinical evidence that IAV and RSV enhance the severity of OM (Heikkinen et al., 1999).

It is becoming increasingly apparent that defects in the host innate immune response of the middle ear drive prolonged inflammation, reduce pathogen clearance, and underlie chronic and recurrent forms of OM. This review explores the innate immune response to both viral and bacterial pathogens in OM, and current knowledge regarding the dysregulation of the host immune response that may underlie the development and recurrence of OM. Animal experimental models, in addition to clinical studies, are discussed, since investigating immune responses to pathogens in isolation in the middle ear is difficult, and mechanistic studies using a range of animal models are essential. However it needs to be noted that murine immune responses may differ to those of humans and so need to be viewed in the context of complementary studies (Bhutta, 2012; Tyrer et al., 2013). A focus of this review is the host innate immune response to NTHi, as the most common otopathogen used to investigate host immune mechanisms using animal models for OM. In contrast, respiratory virus activation of host innate immune responses in OM pathogenesis is less clear, with most fewer animal model studies focussed on the role of IAV and RSV.

## Pathogen Recognition and Activation of Signalling Pathways Within the Innate Immune Response

Within the middle ear, like other mucosal immune locations, molecular signatures known as pathogen-associated molecular patterns (PAMPs), are produced by both bacterial and viral pathogens and recognised *via* one or multiple microbial pattern recognition receptors (PRRs) (Medzhitov, 2007; Kawai and Akira, 2009; Li and Chang, 2021). These receptors are located throughout the middle ear epithelium, and are expressed on the cell surface, internal cell membranes and within the cytoplasm of structural epithelial and antigen presenting cells within the host innate immune system (Leichtle et al., 2011; Kumar et al., 2013; Kurabi et al., 2016).

**Figure 1:**
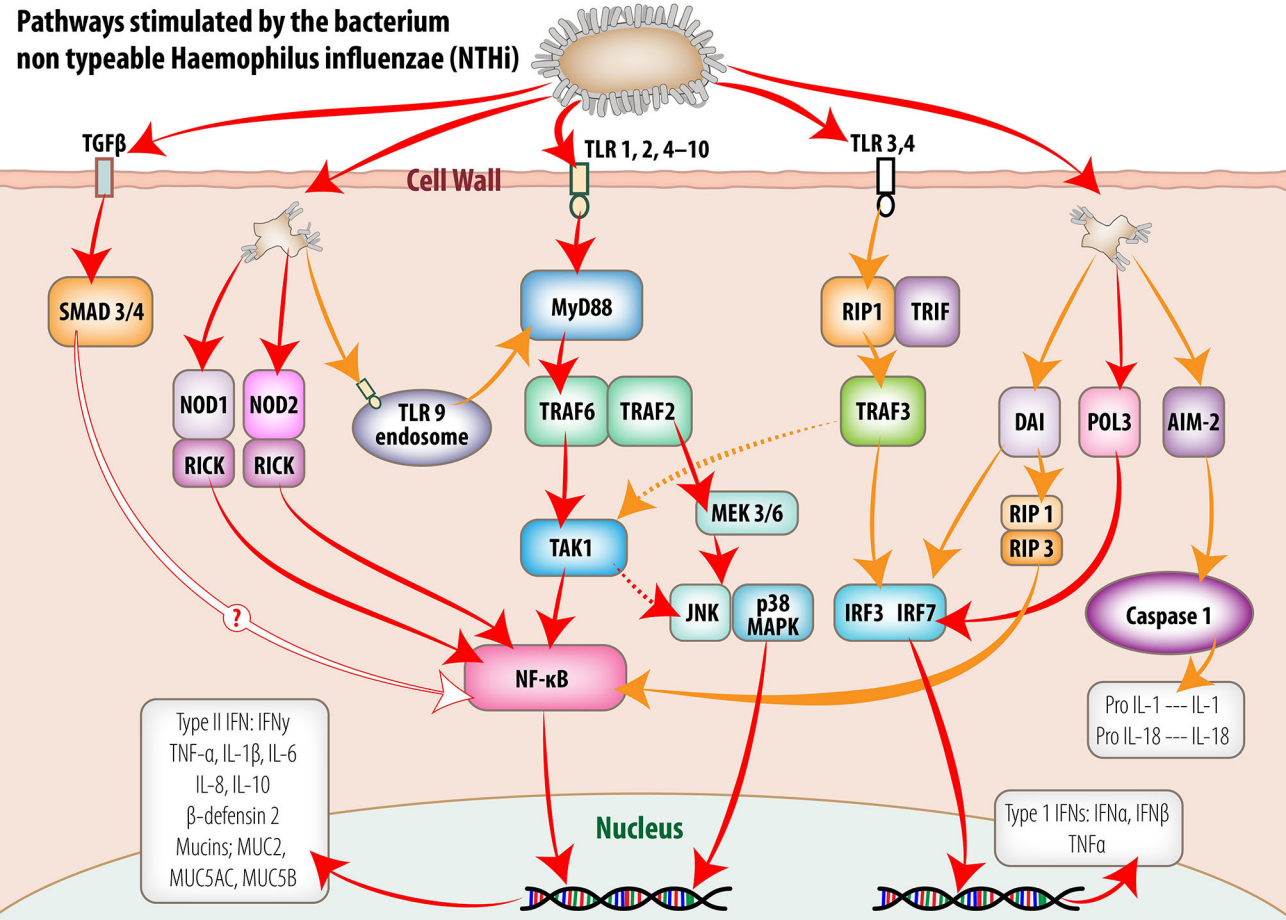
Intracellular signaling pathways utilized by middle ear mucosa in response to stimulation by non-typeable *Haemophilus influenzae* (NTHi). Pattern recognition receptors (PRR) detect conserved molecular signatures of invading microbes and facilitate activation of synthesis and secretion of downstream cytokine cascades. This figure schematically represents the signaling pathways activated by NTHi both at the cell membrane and within the cell cytoplasm of the middle ear mucosal cells. The information portrayed was extracted from published reports of experiments utilizing middle ear cell lines and middle ear epithelium and mucosa from animal models and human biopsy material. (Reproduced, with permission, Massa et al., 2015).

Non-self-DNA and RNA sensing within the middle ear mucosa can involve multiple PRRs depending on the invading pathogen and intrinsic host PRR expression. Overall, this activation of multiple PRRs during the initial innate immune response to pathogens provides increased opportunity for multiple synergistic and/or redundant signalling pathways to be activated, often through NF-κB, interferon response factors (IRFs) and AP-1 transcription factor activation (see **Figure 1**). The efficacy of different signalling pathways can influence host susceptibility to disease (Skevaki et al., 2015). Signalling regulation by host and pathogen factors within the middle ear during OM may lead to modification of timing and secretion of various cytokines, chemokines, interferons, and antimicrobial peptides from the mucosal epithelium or cells recruited to the site of activation. Importantly, the concentrations of antimicrobial peptides and proteins, and cytokines within MEF of children experiencing recurrent AOM are known to increase (Seppanen et al., 2019).

Host innate immunity is activated in response to a wide range of bacterial and viral pathogens through transcription factors downstream from the PRRs and can vary to provide stimulus-specific responses (Borghini et al., 2018). This regulation of the innate immune response is essential to optimise pathogen clearance whilst minimising local tissue damage from inflammatory processes (Massa et al., 2015). For example, PRR activation of the NFκB transcription factor cascade by NTHi increased surfactant protein A (SP-A) expression in SP-A knockout mice, causing more severe inflammation. In contrast, wild-type mice showed improved bacterial aggregation, killing and macrophage clearance of the middle ear due to SP-A modulation of the host inflammatory response (Abdel-Razek et al., 2019).

Thus, differences in host PRR expression and subsequent signalling pathway activation can lead to differences in how the innate immune response is regulated and how OM disease progresses. There is growing evidence that host genetic polymorphisms in PRR genes and differences in PRR expression can dysregulate innate immune signalling pathways in response to otopathogens and therefore play a significant role in the induction and pathogenesis of OM (Lin et al., 2017). Furthermore, NTHi is recently reported to utilise phase variable epigenetic regulation to modify and adapt its phenotype but also modify host immune responses (Robledo-Avila et al., 2020), creating a shifting immunological target for the host (Parrish et al., 2019).

## PRR Activation and Innate Immune Signalling in Response to Bacterial Otopathogens

Toll-like receptor (TLR)-dependent activation of innate immune responses within the middle ear are currently best described from animal, particularly murine models. In these models, the cell surface-expressed TLRs, TLR-2 and TLR-4, play an important role in sensing and responding to bacterial otopathogens (Kweon et al., 2006; Moon et al., 2007; Trune and Zheng, 2009; Kim et al., 2010; Leichtle et al., 2011; Mittal et al., 2014b; Zhang et al., 2015). The overall effect TLR activation is to induce NF-κB signalling, resulting in mucosal hyperplasia and upregulation of pro-inflammatory cytokines (Lim et al., 2007) (see **Figure 1**). Thus, TLRs initiate and mediate expression of a variety of molecules from the middle ear mucosa including inflammatory cytokines (IL-1α, IL-1ß, IL-6, IL-10, TNFα, vascular endothelial growth factor (VEGF), chemokines Ccl3 (macrophage inflammatory protein 1a or MIP1a), Cxcl2 (macrophage inflammatory protein-2 or MIP2), keratinocyte-derived chemokine (KC or Cxcl1; recruits and activates macrophages) and antimicrobial peptides such as mouse ß-defensin 2 and mucin genes (MacArthur et al., 2011; Trune et al., 2015; Kurabi et al., 2016). Interestingly, in association with changes in epithelial cell structure from pseudostratified ciliated columnar epithelium within the eustachian tube to a more squamous epithelium within the middle ear of the rat (Massa et al., 2015), more TLR2 and TLR4 receptor mRNA is expressed within the middle ear compared to other upper respiratory tract locations including the Eustachian tube, nasopharynx and oral cavity (Song et al., 2009). The essential role of TLR2 and TLR4 receptors and signalling molecules, MyD88 and TRIF in the activation of innate immune responses has also been demonstrated using knock-out murine models (Hernandez et al., 2008; Leichtle et al., 2009). Gene deletion of these receptors and signalling molecules results in persistent OM, in the form of thickened mucosa, delayed neutrophil and macrophage recruitment and reduced efficiency of bacterial killing and clearance from the middle ear (Leichtle et al., 2009; Leichtle et al., 2011; Underwood and Bakaletz, 2011; Kurabi et al., 2016).

The importance of cell surface TLR signalling in the innate immune response in OM is best demonstrated in a murine model in which heat-killed NTHi was used for trans-tympanic inoculation (MacArthur et al., 2006; Trune et al., 2015). In these mice, there was no active infection within the middle ear, although an inflammatory response was still induced in response to endotoxins, which act as PAMPs. Within 6 hrs, genes for MIP-1α, MIP-2α, IL-6 and Cxcl1 demonstrated significantly elevated expression (>50->1000 fold) whilst IL-1α (Ccl3), IL-1ß, IL-10, TNFα expression was more moderately upregulated (>4->36 fold). Expression of each of these genes returned to non-stimulated levels by 72 hr in the absence of active infection. Cytokine and chemokine production was increased in parallel with the gene upregulation, however the production of these proteins lagged behind upregulated gene expression by peaking 24hr after inoculation. In this model, TLR4 and TLR9 expression were also significantly increased, while TLR2 expression was only slightly increased (Trune et al., 2015). Interestingly, NTHi infection also increased Chemokine CXC receptor 4 expression in the mouse middle ear model, signalling the inflammatory response through IKKα and p38MAPK pathway activation (Ma et al., 2018). Studies using bacterial otopathogens other than NTHi are limited, however, it has been demonstrated in a mouse pneumococcal OM model that TLR2 expression and NF-κB signalling in the middle ear mucosa, is critical for the recruitment of macrophages. The absence of TLR2 expression resulted in impaired *S. pneumoniae* clearance from the middle ear and a prolonged inflammatory response (Komori et al., 2011; Huang et al., 2016).

Observations regarding the importance of TLR2 in the innate immune response in murine models of OM is supported by clinical studies. In children, TLR2 is a predominant NTHi receptor within middle ear epithelial cells and activation of this receptor leads to the induction of ß-defensin 2 through activation of the MyD88-IRAKI-TRAF6-MKK3/6-p38 MAP kinase signal transduction pathway (Lee et al., 2008). Increased mRNA expression of TLR4 and TLR2 has also been reported within the middle ear fluid (MEF) of children experiencing AOM, with TLR9 expression remaining unchanged. Coincident with the TLR upregulation, mRNA expression of cytokines including, pro-inflammatory TNFα, IL-6, IL-8, IL-10, IL-1ß and chemokines CCL2, CCL3, CCL4, CCR5 and CXCR3 were significantly higher (8-330 fold) in bacterial culture positive MEF samples, and increased mRNA expression of these molecules was associated with increased numbers of bacterial species identified within the sample (Kaur et al., 2015).

In addition to cell surface expressed TLR signalling, endosomal TLR9 was shown to be important in sensing NTHi when inoculated into the murine middle ear. Deletion of TLR9 from mice prolonged middle ear inflammation and bacterial clearance (Leichtle et al., 2012). In this same study, Leichtle et al, also identified non-TLR pathogen DNA sensing genes to be upregulated in response to infection by NTHi. These included DNA-dependent activator of IFN regulator factor (DAI), absent in melanoma-2 (AIM2) and Pol-III in addition to other genes encoding proteins that mediate downstream signalling pathways (Leichtle et al., 2012).

Several other families of PRRs contribute to PAMP recognition and activation of the signalling pathways to activate and modulate innate immune responses, including retinoic acid-inducible gene 1 (RIG-I)-like receptors (RLR’s), cytoplasmic nucleotide-binding oligomerisation domain (NOD)-like receptors (NLRs) and DNA sensing receptors (Kurabi et al., 2016). Compared to surface expressed TLR receptors, less is known concerning the importance of cytoplasmic PRRs in sensing and responding to bacterial infections in the middle ear (Kumar et al., 2011; Kurabi et al., 2016). However cytoplasmic NOD-like receptors and RIG-1 receptors exhibit reduced expression in patients with OM which may be associated with the development of recurrent OME (Kim et al., 2014). NOD1/NOD 2 receptors activate innate immune responses during OM to reduce infection (Lee et al., 2019). More specifically, NOD2 mediated β-defensin 2 regulation is activated after NTHi penetration of the cell membrane and helps to prevent OM development (Woo J. I. et al., 2014). Activin-like cell surface receptor kinases and serine/threonine kinases also activate important signalling pathways in the pathogenesis of OM. These receptors lead to the activation of TGF-β, which is a pleiotropic cytokine and a key regulator of tissue remodelling.

Although several murine models of bacterial OM have demonstrated an upregulation of pro-inflammatory responses to infection within the mucosa, the entire process of immunoregulation during OM may be more complex. A report on the transcriptome signature elicited from PBMCs at the onset of AOM in children caused by NTHi reported that genes associated with antibacterial activity and cell-mediated immunity were predominantly affected. Importantly, the study suggested that NTHi infection suppressed more immune responses than were activated. More specifically, 90% of genes associated with pro-inflammatory cytokine responses were down-regulated, as was classic complement pathway activation (Liu et al., 2013). Furthermore, the transcriptome of a complete episode of NTHi-induced AOM in a mouse model was examined *via* expression profiling utilising whole genome microarrays in the murine model. Sets of genes involved in activation of the innate immune response, negative regulation of that response, epithelial and stromal cell marker changes and neutrophil and macrophage recruitment and function were identified. Overall, positive and negative regulation of inflammatory processes were recognised, and the importance of anti-inflammatory responses in control of OM pathogenesis were highlighted (Hernandez et al., 2015). Importantly, regulation of NTHi triggered activation of inflammatory responses by natural products such as the plant pigment Quercetin may provide potential therapeutic approaches to reduce OM (Ma et al., 2018).

## Virus-Induced Innate Immunity

Respiratory viruses alone can induce AOM and lead to chronic presentations of OM (Heikkinen and Chonmaitree, 2000). A causal relationship between viral URTI and eustachian tube obstruction, middle ear pressure and bacterial colonisation has been demonstrated in both human challenge studies using rhinovirus (McBride et al., 1989; Buchman et al., 1994) and IAV (Doyle et al., 1994) and in both chinchilla and mouse models of AOM induced by IAV (Giebink et al., 1987; Short et al., 2011). In addition, viral-bacterial coinfections have demonstrated more severe outcomes for AOM in humans (Moore et al., 2010; Binks et al., 2011; Pettigrew et al., 2011; Chonmaitree et al., 2016; Schilder et al., 2016) and animal models (Giebink and Wright, 1983; Patel et al., 1992; Brockson et al., 2012), compared to bacterial colonisation alone. However, despite this clear link between respiratory virus infections and OM, the exact mechanisms by which viruses promote the development of AOM and subsequent chronic and recurrent OM is not well known due to a paucity of studies on the immune response during virus-only AOM. Therefore, our understanding of the role of respiratory viruses in the immune response during OM is based largely on our general understanding of the immunopathology of URTIs, which affect the nasopharynx foremost with AOM considered a secondary complication (Heikkinen, 2000; Chonmaitree et al., 2008; Bakaletz, 2010; Kalu et al., 2011; Chonmaitree et al., 2016).

There are few human clinical studies that have sought to characterise the immune response to respiratory viruses during OM and understand how this contributes to pathogenesis of disease. One study collected sera from 145 children with AOM and found that RSV-associated AOM correlated with elevated serum concentrations of proinflammatory cytokines (Patel et al., 2009a). These included G-CSF, MCP-1, IL-10, IL-6, IFN-γ and IL-8, with G-CSF concentrations predicting RSV-associated OM with 87% accuracy. In this study, local mucosal responses were not investigated, although in a related study, the cytokines detected in nasopharyngeal secretions of 326 children with virus-positive URTIs were identified and correlated with virus identification and the onset of AOM (Patel et al., 2009b). All viruses detected (adeno-, entero- rhino- viruses, RSV, Parainfluenza viruses, Influenza viruses) induced significant quantities of IL-1β, IL-6 and TNFα. However only IL-1β was significantly associated with the onset of AOM and was not correlated with any particular virus.

Some studies have identified innate immune responses of cultured human middle ear epithelial (MEE) cells when infected with IAV. A transcriptional study of cells up to 24h post infection revealed upregulation of genes encoding an array of type I interferon inducible signalling pathways, transcription factors, cytokine and chemokine genes, as expected in response to viral infection (Tong et al., 2004). MEE cells also secrete elevated levels of MIP-1α and MIP-1β, TNF-α, IL-6, IL-8 and IL-10 in response IAV infection (Tong et al., 2003; Tong et al., 2004). Interestingly, when IAV strains were compared, H3N2 IAV induced a stronger IL-6 and IL-8 response by MEE cells in culture than H1N1 (Short et al., 2013).

These human studies are informative regarding antiviral responses, although do not provide mechanistic detail regarding how the immune response to viral infections drives the development of OM. Animal models have been used to understand the role of viral URTIs in the development of OM. However, very few have focused on the induction of AOM by viruses alone, without bacterial involvement. Early chinchilla models demonstrated clinical signs and symptoms of OM induced by viruses alone (Giebink et al., 1987), including general observations of tympanic membrane inflammation (Giebink and Wright, 1983). Short et al. (2011) developed the first infant murine model of IAV-induced AOM (Short et al., 2011), where IAV replicated in the middle ear epithelium, and caused submucosal edema and an influx of immune cells, predominantly neutrophils, into the middle ear cavity. These mice also displayed hearing loss indicative of clinical AOM. In a follow up study, inoculation of mice with recombinant IAVs identified a pro-inflammatory response in the middle ear, specific to replicating viruses with a H3-type HA attachment viral protein. This response was dominated by upregulation of genes expressing IL-1β, IL-1α and CXCL2 in the middle ear (Short et al., 2013).

## Innate Immune Responses to Polymicrobial Infections

Adding to the complexity of the host innate immune responses, microbe to microbe interactions are being recognised within the upper respiratory tract microbiome (Schenck et al., 2016) which may enhance or impair bacterial competition and the host innate immune response. For example, peptidoglycan secretion by *H. influenzae* can activate neutrophils to enhance complement-dependent killing of *S. pneumoniae* (Lysenko et al., 2005), whilst *S. pneumoniae* can impair NTHi evasion of host immune responses (Shakhnovich et al., 2002). Furthermore*, S. pneumoniae* and NTHi can synergistically upregulate TLR2 expression, increasing inflammation (Ratner et al., 2005).

Most mechanistic studies in animal models of OM have used single infections to understand cause and effect. However, polymicrobial animal models for OM more closely replicate the complex microbial environment of the nasopharynx and infected and/or inflamed middle ear (Bakaletz, 2010), and may better reflect COM pathogenesis (Holder et al., 2012) and immunisation responses (Novotny et al., 2017). A rat model of OM involving dual infection of the middle ear with NTHi and Pneumococcus type 6A (Pn6A) demonstrated upregulation of genes encoding inflammatory Th2 cytokines and effectors of the TGF-β signalling pathway, which resulted in pathogenic changes and thickening of the mucosa and submucosal layers of the middle ear during OM (Lee et al., 2011). It has been demonstrated in chinchilla that middle ear infection with NTHi promotes the persistence of *M. catarrhalis via* the formation of polymicrobial biofilms (Armbruster et al., 2010). In a mouse model, *M. catarrhalis* impacted pneumococcal OM more than NTHi regarding bacterial load, incidence rate and persistence of infection. Nitric oxide was measured as an indication of inflammation and was elevated in polymicrobial infections significantly more than single infections. Interestingly, pre-infections with the respiratory virus, Sendai Virus, enhanced bacterial OM for all three otopathogens, demonstrating the importance of viral infection in the development of bacterial OM (Krishnamurthy et al., 2009).

Most animal models of virus-induced OM have investigated the role of viruses in supporting subsequent bacterial colonisation of the eustachian tube and middle ear. The chinchilla model has been the most utilised to study the pathogenesis of viral-bacterial co-infection in OM (Giebink and Wright, 1983; Suzuki and Bakaletz, 1994). However, it does have limitations, in that the immune responses and the presentation of AOM in these animals can vary depending on the order and timing of infection and partnering of viruses and bacteria. These variables have made it difficult to build an accurate picture of the role of antiviral innate immune responses in AOM. It has also been difficult to identify specific mechanisms by which viral dysregulation of the innate immune response promotes bacterial colonisation. Therefore, most studies report general inflammation and clinical signs of OM on tympanometric investigation of animals.

Murine models of IAV and *S. pneumonia* co-infection have demonstrated more severe AOM than single infections of either virus or bacteria alone, including greater hearing loss, and middle ear inflammation (Short et al., 2013), in addition to reduced ciliation, hyperplasia of the mucosal epithelium and increased goblet cells (Tong et al., 2014). Tong et al. (2104) also identified that the anaphylatoxins C3a and C5a were expressed in both serum and middle ear lavage from IAV-infected mice indicating that induction of the complement alternative pathway reduced bacterial clearance and enhanced the severity of acute pneumococcal OM. When paired with *M. catarrhalis* and NTHi in experimental Chinchilla infections, RSV was associated with clinical signs of inflammation and haemorrhagic foci in the middle ear mucosa (Brockson et al., 2012), thus compromising the ability of the mucosa to combat ascending bacterial infections and biofilm formation in the middle ear. The chinchilla model has also been useful in demonstrating the ability of different viruses in enhancing bacterial OM. Type 5 adenovirus (Ad5) has been shown to promote infection of the middle ear by *S. pneumoniae* (Murrah et al., 2015), while type 1 adenovirus (Ad1) has been shown to promote ascension of NTHi (Suzuki and Bakaletz, 1994) although not *S. pneumoniae* (Tong et al., 2000) or *M. catarrhalis* (Bakaletz et al., 1995). It is likely that the role of viruses in OM is complex and involves not only inflammation and innate immune dysregulation, but also other factors that enhance bacterial colonisation such as epithelial damage, mucus production and modulation of antimicrobial peptides, as has been demonstrated within other compartments of the respiratory mucosa (Melvin and Bomberger, 2016).

## Antimicrobial Factors Released by the Mucosal Epithelium

In addition to classical innate and adaptive immune responses, the production of anti-microbial molecules, mucus secretion, and mucociliary clearance within the middle ear and eustachian tubes, work in combination to maintain the relative sterility of the middle ear cavity (Lim et al., 2000; Massa et al., 2015). Morphologically, the middle ear region adjacent to the eustachian tube and the eustachian tube itself, exhibit cellular characteristics shared by other mucosal surfaces within the upper respiratory tract, such as a secretory, pseudostratified, and ciliated columnar epithelium (Lim, 1976; Lim, 1979; Bluestone and Doyle, 1988; Martin et al., 2017). The physical resilience of the epithelium is reinforced *via* tight junctions (Tsukita et al., 2008; Yonemura, 2011) and goblet cells that secrete mucus to provide a barrier to adherence and colonisation by bacteria, (Linden et al., 2008), and contribute to the mucociliary clearance of the tympanic cavity and eustachian tube (Evans and Koo, 2009; Martin et al., 2017).

Most importantly, the mucosal response includes activation of antimicrobial molecules, such as lysozyme, ß-defensins and lactoferrin which either independently or together, act to inhibit bacterial colonisation and activate the adaptive immune response (Underwood and Bakaletz, 2011). The significance of antimicrobial molecules has been demonstrated using animal models. For example, lysozyme knockout mice exhibit increased susceptibility to bacterial colonisation of the middle ear and enhanced inflammatory response to *S. pneumoniae* 6B infection (Shimada et al., 2008). Furthermore, in humans, lysozyme and ß-defensin 2 can synergistically partner to directly kill invading *S. pneumoniae* 6B (Lee et al., 2004) and protect against NTHi induced OM (Woo J. I. et al., 2014). Most recently, a study examining middle ear effusate from children experiencing recurrent AOM confirmed the importance of elevated antimicrobial protein (AP) and cytokines as potential markers for bacterial persistence and inflammation (Seppanen et al., 2019). Antimicrobial proteins or host defence peptides may offer future treatment options against polymicrobial infections (Bergenfelz and Hakansson, 2017; Batoni et al., 2021).

Viruses, conversely, have been shown to enhance bacterial infection of the middle ear by suppressing antimicrobial factors and enhancing bacterial adhesion molecules. McGillivary et al. (2007) demonstrated that both viruses and bacteria can alter the expression of cationic APs in the upper airways, thus enabling an expansion of otopathogens. This elegant study involved the expression of recombinant cCRAMP, a cathelicidin homolog from the upper respiratory tract of the chinchilla in cultured chinchilla middle ear epithelial cells (CMEEs). CMEEs were infected with IAV, RSV or Ad1, and the effect on cCRAMP and also cBD-1, which is the murine ortholog of the human AP β-defensin, was investigated (McGillivary et al., 2007). IAV reduced cCRAMP mRNA expression by 50%, while RSV and Ad1 only had a minimal effect. In contract RSV reduced the expression of cBD-1 mRNA by 40%, while IAV and Ad1 did not. Thus, highly otopathic viruses, such as IAV and RSV appear adept at differentially reducing key antimicrobial defence molecules in airway epithelial cells using different mechanisms. The ability of RSV to suppress cBD-1 expression has also been demonstrated directly in a chinchilla RSV/NTHi co-infection model. In this model, RSV intranasal challenge diminished both cBD-1 mRNA and protein expression in the upper airway epithelium. Consequently, when chinchilla were inoculated with NTHi, there was a marked increase in both the quantity and duration of NTHi recovered from the nasopharynx (McGillivary et al., 2009). These studies provide evidence that virus-induced dysregulation of AP expression contributes to the elevated bacterial colonisation to precedes AOM (Bakaletz, 2010).

The net effect of viral URTI is a reduction in the protective function of the mucosal epithelium, which extends from the nasopharynx through the eustachian tubes and into the middle ear. This compromise in protection renders the middle ear susceptible to ascending bacterial infection, and can last from 2-10 weeks in children before homeostatic conditions are returned (Carson et al., 1985).

## Immunocompetent Cell Types Within the Mucosal Epithelium

The healthy middle ear of both humans and animal models of OM is host to immunocompetent cell types, although not in significantly high numbers until infiltration occurs during AOM (Jecker et al., 1996; Jecker et al., 2001; Suenaga et al., 2001) or COME (Enoksson et al., 2020). Mast cells and macrophages are the predominant cell types in non-inflamed middle ear, with dendritic cells and macrophages present in the tympanic membrane (Ichimiya et al., 1990; Mittal et al., 2014b) These antigen-presenting cell phenotypes provide a mechanism for interconnection of the local innate mucosal immune response with the more specific adaptive immune system (Ichimiya et al., 1997). Enhanced inflammation within the middle ear mucosa, stimulated through pro-inflammatory cytokine secretion including ß-defensin 2 and other defensins (Underwood and Bakaletz, 2011) and pre-inflammatory molecules including TNFα, IL-1β and CCL3, attract other immunocompetent cells *via* chemotaxis, including dendritic cells, memory T-cells, neutrophils and mast cells within the epithelium (Yang et al., 2007). In addition to direct bacterial killing, ß-defensin 2 contributes to the mediation of adaptive immunity (Yang et al., 2007; Underwood and Bakaletz, 2011).

Upregulation of the innate immune response, as evidenced by immunocompetent cell infiltration, has long been demonstrated in COM patients (Palva et al., 1981; Enoksson et al., 2020), whilst mouse models of NTHi infection of the middle ear have also demonstrated leukocyte infiltration (Hernandez et al., 2008; Hernandez et al., 2015; Trune et al., 2015). Furthermore, the acute inflammatory response during AOM in the rat model shows time-dependent increases in identification of macrophages, dendritic cells, polymorphs and natural killer cells (Jecker et al., 1996; Forseni et al., 1999), with T-cells present 3 days after perforation of the tympanic membrane (Tahar Aissa and Hultcrantz, 2009).

The activation and rapid response of the innate immune response within the middle ear mucosa is well evidenced from animal studies, particularly mouse models (Bhutta, 2012). Evidence of the precise timing and duration of immunocompetent cell changes occurring in the murine middle ear demonstrates that neutrophil recruitment is evident within 6 hrs of inoculation of middle ear and peaks 24 hours post inoculation at level ~10-fold greater than the peak recruitment of macrophages observed at 48 hours after inoculation (Leichtle et al., 2011). The number of neutrophils within the ME epithelium returned to control levels after 72h, whereas macrophages were still observed 120hr post inoculation. Epithelial effusion within the middle ear is present 24-48hr post inoculation, consistent with peak neutrophil and macrophage (Hernandez et al., 2015) recruitment. Unfortunately, increased neutrophil recruitment can be utilised by NTHi to avoid phagocytosis through host formation of neutrophil extracellular traps (Juneau et al., 2011; Schachern et al., 2017) which have been identified within the MEF of children with OM (Thornton et al., 2013).

Neutrophil infiltration of the middle ear is also a feature of IAV-induced OM in a murine model (Short et al., 2011). IAV also depressed polymorphonuclear leukocyte chemiluminescence activity in a chinchilla model (Giebink and Wright, 1983). Interestingly H3N1 had a more significant effect on this response than H1N1, similar to the findings of Short et al. (2013).

## Innate immune response activation of the adaptive immune response

Overall, there is a paucity of human studies, however, many murine studies have explored the interconnection of the innate immune responses occurring within the middle ear and their role in activation of the adaptive immune response. Within 3 days post inoculation with type B *Haemophilus influenzae*, macrophages and Mac-1+ neutrophils were present in the middle ear of mice whilst within the middle ear mucosa, Lyt-1+ T cells and Lyt-2+ T suppressor/cytotoxic cells were present 7 and 14 days post inoculation. The majority of mucosal immunoglobulin-bearing cells at Day 14, were IgA+ lymphocytes although IgG+ and IgM+ T –cells were present 3, 7 and 14 days post-inoculation. Lyt-1/L3T4+ T lymphocytes were present in larger numbers than B lymphocytes thus helper T cells appear to have significant involvement in AOM (Krekorian et al., 1991). Recent profiling of the cells within the murine middle ear using single-cell transcriptomics identified 17 different cell clusters, reflecting different cell types within the normal middle ear however tissue monocytes appear to have a primary role in regulation of acute middle ear infection response in the complex innate defence (Ryan et al., 2020). Interestingly, adaptive immune system factors such as TBX21, a transcription factor, may also participate in innate immune response regulation during *S. pneumoniae* infection, through modifying TLR2 expression (Woo C. H. et al., 2014). Whilst these model studies establish a link between innate and adaptive immune responses in the middle ear, additional studies are needed to confirm such links in the human middle ear.

Recent evidence highlights the complexity of the upregulation of specific inflammatory cells and mediator secretion varies in response to the microbial otopathogens involved and the child’s gender, with *Moraxella* and *Haemophilus* species tending to stimulate more inflammatory mediators in the middle ear (Enoksson et al., 2020). Interestingly, within the few human studies available, reports of relationships between IgG, IgG1, IgG2 and IgA are complicated by case-definitions of the sampled population (Corscadden et al., 2013). A well-designed cross-sectional study of serum and middle ear levels of IgG, IgG1 and IgG2 in children under 3 years of age, with and without a history of frequent AOM reported that of 11 different pneumococcal serotypes present, only serotype 5 induced elevated serotype specific IgG and IgG1. This study evidenced that frequent AOM is not the result of deficiency in IgG, IgG1 or IgG2 response (Corscadden et al., 2013). Further research, focussed on both viral and bacterial otopathogens are needed to the confirm the innate activation of the adaptive immune responses of children experiencing OM.

## Host Factors That Modulate the Mucosal Response in OM

In this review we have explored the innate immune response to predominant otopathogens in the context of OM pathogenesis. Induction of a productive innate immune response that will clear otopathogen infection, whilst minimising inflammation is ideal for AOM resolution, while a hyperinflammatory response and upregulation of bacterial adhesion mechanisms is deleterious to this process and may result in recurrent or chronic forms of OM. Otitis media susceptibility is associated with a number of host genes, identified in previous reviews (Kurabi et al., 2016; Bhutta et al., 2017; Geng et al., 2019). Genetic factors that influence the heritability of susceptibility to OM are reported to range between 40%-70%, based on several prospective, longitudinal prospective and retrospective twin studies (Mittal et al., 2014a). In humans, reduced expression of key PRRs TLR9, NOD1, NOD2 and RIG-I has been observed in the MEF of OM-prone compared to non-OM-prone children (Kim et al., 2010). In addition, TLR2, IL-1, IL-6 and TNFα gene and protein production were reduced in children aged 2-7 years compared to 0-2 and over 7-year olds, and were also lower in culture-positive OM (Kim et al., 2015). Interestingly, in contrast, one report identified no significant differences in mRNA and protein levels of TLR2, TLR4 and TLR5 between non-OM and chronic OM patients, however the levels fell for CSOM patients. These studies indicate a correlation between reduced PRR function and severity of disease (Si et al., 2014).

This lack of functionality in PRR signalling and subsequent protective innate immune responses in OM-prone individuals is likely due to polymorphisms in candidate genes related to innate and adaptive immunity. Children identified with TLR2 and TLR4 polymorphisms exhibit disrupted innate immune responses that increase their susceptibility to OM (Hafren et al., 2015; Toivonen et al., 2017). Genetic polymorphisms in Mannose-binding lectin and TLR2,3,4,7 and 8 can promote or protect children from the risk of respiratory infections and AOM (Toivonen et al., 2017) indicating that we do not fully understand the mechanisms of innate immunity influenced by gene polymorphisms.

Allelic association studies have identified polymorphisms related to the development of OM in genes encoding TLR4 receptors, IL-6, IL-10 and TNF-α (Emonts et al., 2007), mannose-binding lectins (Wiertsema et al., 2006), surfactant (Ramet et al., 2001), and Mucin gene MUC5AC (Ubell et al., 2010) [for review see (Mittal et al., 2014a; Lin et al., 2017; Geng et al., 2019)]. TLR4 locus polymorphisms have also been examined in patients with RAOM or COME and demonstrated a role for TLR4 in the regulation of the innate immune response. The inability of this study to replicate the association of a previously unrecognised TLR4 haplotype in two independent Finnish cohorts with UK or 2 US cohorts emphasises the potentially heterogenous nature of OM and the complexity of environmental and host factors that may modulate overall susceptibility to OM (Hafren et al., 2015; Einarsdottir et al., 2016). Polymorphism IL-1β+3953 is associated with more severe presentations of AOM and has been associated with higher risk of severe inflammation post-AOM infection (McCormick et al., 2011). Recently, the potential impact of cohort heterogeneity and the complexity of environmental and host factors on OM development has been reviewed for a unique “stringently-defined otitis-prone” population, where microbial confirmation of AOM through tympanocentesis was undertaken. The children in this cohort demonstrate a wide range of dysfunctional innate and adaptive immune responses that increase their vulnerability to upper respiratory tract infections and OM (Pichichero, 2020).

## Conclusion

The middle ear clearly demonstrates the structural and functional features of a mucosal immune site, that for some children, results in increased susceptibility to recurrent or chronic OM. Investigations using a range of animal models (chinchilla, rat, mouse) and particularly genetically modified knockout mice, continue to improve our insight and understanding of activation and regulation of the host-microbial interactions within the middle ear in response to a variety of pathogens. The cellular immune apparatus within the middle ear can mount a rapid innate immune response to invading pathogens. Ongoing investigation and characterisation of the innate and adaptive immune responses of the middle ear mucosal tissues using clinical studies and animal models will elucidate this region’s response to individual and multiple otopathogen infections, and the host factors that influence OM disease progression or clearance and response to vaccination. Some pathogens, such as NTHi can activate multiple, overlapping induction pathways resulting in innate immune system upregulation. Co-infection with multiple pathogens can also influence the host response. It is increasingly recognised that genetic polymorphisms, the level of expression of key regulatory molecules for the innate immune response and environmental factors such as pathogen load may impair innate immune system function and substantially increase the risk of predisposition of some children to more frequent or severe OM. Future studies need to explore the genetic and environmental interrelationships of these polymorphisms and their impact on predisposition or susceptibility to OM and its severity. Next generation sequencing and large, well-phenotyped populations from multiple regions will expand the applicability of these data to support development of new treatment strategies that may enhance innate immunity such as monophosphoryl lipid A (MPL) studies in mice (Iwasaki et al., 2017), β-defensin 2 and probiotics or alter the activation of the innate immune responses to reduce inflammation and chronic OM pathogenesis.

## Author Contributions

All authors contributed to the concept and writing of this review and agree to publication.

## Conflict of Interest

The authors declare that the research was conducted in the absence of any commercial or financial relationships that could be construed as a potential conflict of interest.

## Publisher’s Note

All claims expressed in this article are solely those of the authors and do not necessarily represent those of their affiliated organizations, or those of the publisher, the editors and the reviewers. Any product that may be evaluated in this article, or claim that may be made by its manufacturer, is not guaranteed or endorsed by the publisher.
